# Congenital Diaphragmatic Hernia Repair in a Patient With Pallister-Killian Mosaic Syndrome and Left Ventricular Hypoplasia

**DOI:** 10.7759/cureus.23095

**Published:** 2022-03-12

**Authors:** Piotr Kostyk, Irim Salik

**Affiliations:** 1 Anesthesiology, Westchester Medical Center, Valhalla, USA; 2 Anesthesia, Westchester Medical Center, Valhalla, USA

**Keywords:** pulmonary hypertension, craniofacial dysmorphism, left ventricular hypoplasia, pallister-killian syndrome, congenital diaphragmatic hernia

## Abstract

We present the case of a two-week-old infant with congenital diaphragmatic hernia (CDH) and Pallister-Killian mosaic syndrome (PKS) for CDH repair. We discuss the pathophysiologic findings of both conditions and the resulting anesthetic challenges from their interplay.

## Introduction

Congenital diaphragmatic hernia (CDH) results in varying degrees of pulmonary hypertension or hypoplasia secondary to herniation of abdominal contents into the thoracic cavity. The prevalence is 2.5 cases per 10,000 births and is associated with high mortality rates as well as significant long-term morbidity [1]. Up to 10% of non-isolated cases are associated with genetic syndromes, including Beckwith-Wiedemann, Cornelia de Lange, Denys-Drash, Donnai-Barrow, and Pallister-Killian mosaic syndrome (PKS), to name a few [2]. Known as mosaic tetrasomy 12p, PKS is one of the more common microduplications associated with CDH. An ominous finding in infants with CDH is left ventricular hypoplasia, which can significantly increase morbidity and mortality. We present the complex case of a two-week-old infant with left ventricular hypoplasia, CDH, and PKS for CDH repair.

## Case presentation

We present the case of a two-week-old, 2.8 kg male, born at 34-weeks gestation secondary to oligohydramnios and premature rupture of membranes. The patient presented with neonatal respiratory distress and pulmonary hypertension at birth. Echocardiography revealed dextrocardia, moderate tricuspid valve insufficiency, mild left ventricular hypoplasia, and right ventricular dilatation with a severely increased mean pulmonary artery pressure.

**Figure 1:**
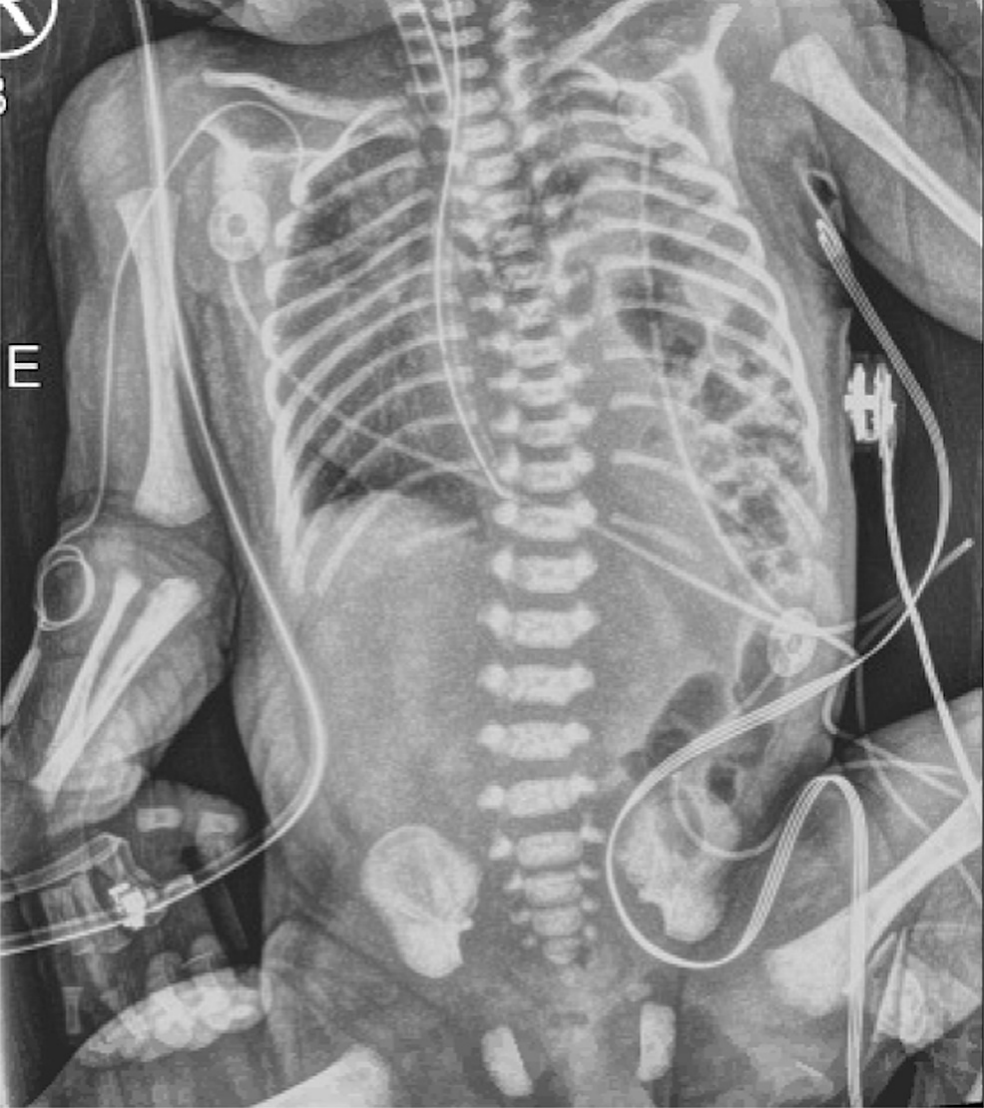
Preoperative anteroposterior radiograph of infant demonstrating a left-sided congenital diaphragmatic hernia with resultant dextrocardia

In addition, the infant presented with numerous dysmorphic facial features, including micrognathia, facial asymmetry, patchy facial hyperpigmentation of the face and scalp, low set ears, and hypertelorism. The infant exhibited rhizomelic limb shortening and supernumerary nipples. PKS was suspected on the basis of the aforementioned phenotypic characteristics and confirmed via cutaneous fibroblast analysis that revealed an extra chromosome. The patient was subsequently diagnosed with a CDH (See Figure 1).

The infant was intubated shortly after birth by the anesthesia team to avoid gastric insufflation during spontaneous respiration. The anesthesia team was contacted due to the potential for difficult intubation secondary to facial asymmetry and micrognathia. The patient was transported to the operating room and induced with sevoflurane inhalation induction and 0.5 mcg/kg dexmedetomidine. A size 1.0 laryngeal mask airway was placed, and the patient was intubated with a 3.0 mm uncuffed endotracheal tube (ETT) via fiberoptic bronchoscopy through the device without significant changes in cardio-respiratory hemodynamics. As the position of the carina is located higher in the larynx in CDH patients, the endotracheal tube was secured 0.5 cm higher to avoid a right mainstem intubation. After successful intubation, an orogastric tube was inserted and placed on low continuous suction. Intravenous access was obtained in the upper extremities. The infant was transported back to the neonatal intensive care unit (NICU) for medical stabilization prior to definitive CDH repair.

At two weeks of age, the patient presented intubated with the 3.0 ETT in situ and was ventilated with pressure control ventilation with 30 cm H2O with resultant tidal volumes of 12 to 16mL, rate of 30 breaths per minute, and the fraction of inspired oxygen (FiO2) 70%. The patient was maintained on inhaled nitric oxide at 20 ppm, and midazolam and fentanyl infusions were continued from the NICU. Two 24G peripheral IVs were present. Before the procedure, an ultrasound-guided radial arterial line was placed. The patient received 6 mcg of fentanyl and 5 mg of rocuronium prior to the start of the procedure. Soon after, a large leak was detected, and the in situ endotracheal tube was replaced with a 3.5 mm un-cuffed endotracheal tube under indirect visualization with a video laryngoscope without incident. Preductal saturation was maintained between 85-95% to avoid both hypoxia and hyperoxia or elevated pulmonary vascular resistance while the partial pressure of carbon dioxide (PaCO2) was maintained between 55 to 70. During mechanical ventilation, the peak inspiratory pressure (PIP) was maintained at < 25 cm H2O to avoid contralateral pneumothorax. Anesthesia was maintained with a high-dose opioid technique, fentanyl 10 μg/kg, and rocuronium 1 mg/kg following re-intubation.

Intraoperatively, the patient became hypotensive to blood pressure 43/22 and required 20 ml/kg packed red blood cell transfusion. The CDH repair was completed without incident and the pre and postductal saturation remained 95% and 96%, respectively, throughout the procedure. Following CDH repair, the infant patient was transported to the neonatal ICU, intubated, and mechanically ventilated with a FiO2 of 1.0 with oxygen saturation of 93% on inhaled nitric oxide (iNO). The patient was hemodynamically stable with no immediate postoperative complications; a tracheostomy was performed on postop day 55. The infant remained on iNO until postoperative day 75, at which time, his estimated right ventricular systolic pressure estimate was only mildly increased, and the tricuspid regurgitation velocity had decreased on trans thoracic echocardiogram when compared to the initial NICU presentation. He was transitioned to sildenafil, hydrochlorothiazide, and spironolactone. A gastrostomy tube was placed on postop day 79 and the patient was discharged to a long-term care facility on postop day 86. Currently, the patient still resides at a skilled nursing facility, requiring mechanical ventilatory support.

## Discussion

There can be extensive pulmonary involvement in infants with CDH, including acinar hypoplasia with reduced or thickened alveoli, and increased interstitial tissue [3]. Pulmonary vascular remodeling can result in irreversible persistent pulmonary hypertension of the newborn (PPHN). Left ventricular (LV) hypoplasia, first described by Siebert et al. [4], leads to reduced cardiac mass, left atrial, LV, and interventricular septal hypertrophy associated with extremely high infant mortality. Dire outcomes are likely due to cardiac rotation leading to reduced blood flow across the foramen ovale, reduced filling of left heart structures, and decreased LV growth [5]. Diastolic dysfunction and LV hypoplasia are frequently implicated in reducing the efficacy of pulmonary vasodilator therapy in CDH patients [6]. Poor prognostic indicators in infants with CDH include cardiac or chromosomal abnormalities, hepatic herniation, lung area/head circumference ratio (LHR) < 25%, percent predicted lung volume (PPLV) < 15%, total lung volume < 20 mL, LV hypoplasia, and right-sided CDH. Infants with a low PPLV had significantly higher rates of extracorporeal membrane oxygenation (ECMO) use [7-8].

A 2016 randomized controlled trial known as Ventilation in Infants with CDH (VICI) [9] found that infants with CDH who are mechanically ventilated conventionally have a shorter ventilation duration, reduced need for inhaled nitric oxide (iNO), sildenafil, vasoactive medications, and ECMO when compared to infants who received high-frequency oscillatory ventilation (HFOV). However, HFOV may still be considered in infants who are difficult to ventilate with a PIP of 30 cm H2O or at a rate of 60 breaths/min [10]. Utilizing HFOV, mean airway pressure (MAP) should be limited to 14-16 cm H2O and the pressure delta (Δ P) should range from 30-40 cm H2O. The initiation of ECMO should be considered if patients cannot be oxygenated with a MAP of 16 cm H2O, preductal saturations less than 85%, postductal saturations less than 70%, PaO2 less than 40 mmHg, and/or an oxygenation index greater than 40 for at least 3 hours [11]. Infants commonly develop an ipsilateral pneumothorax, although this should not be treated unless under tension. Tension pneumothorax should be suspected with unstable hemodynamics, poor oxygen saturation, and a failure of fluid accumulation within the pneumothorax space.

Infants with CDH should undergo urgent echocardiography to diagnose co-morbid congenital heart disease, flow across the patent ductus arteriosus and patent foramen ovale, and an approximate measurement of the right ventricular systolic pressure (RVSP) based upon the tricuspid regurgitation jet [12]. Pulmonary vasodilators are generally more efficacious in infants with supra-systemic RVSP, although these patients are at high risk for LV diastolic dysfunction. Postoperative decompensation is less likely if CDH repair is undertaken following the lowering of PA pressure to <80% of systemic pressure. Milrinone is commonly used in infants with LV dysfunction to enable LV relaxation due to its lusitropic properties. Milrinone has been shown to improve RV function and oxygenation in a small case series of infants with CDH and pulmonary hypertension [13]. In an infant on ECMO, there is conflicting data on optimal timing for CDH repair. The highest postoperative morbidity is associated with late repair on ECMO while early repair may be beneficial in infants who are not able to be weaned.

PKS is a sporadic disorder caused by mosaic tetrasomy of the short arm of chromosome 12 resulting in a number of morphologic abnormalities. Children are commonly afflicted with coarse facies, hypotonia, seizure disorder, psychomotor delay, hearing loss, micrognathia, high forehead, frontotemporal alopecia, hypertelorism, skin changes, and midface malformations, including cleft palate, macroglossia, or prognathism [14]. Additional commonly reported congenital anomalies include diaphragmatic hernia, anal anomalies, renal dysplasia, congenital cardiac defects, neurologic abnormalities, laryngomalacia, gastroesophageal reflux disease, oculo-retinal changes, and hypo- or hyperpigmented skin changes [15]. Patients with PKS may exhibit congenital heart disease, including atrial septal defect, aortic coarctation, aortic stenosis, hypertrophic cardiomyopathy, and pericardia agenesis. Craniofacial dysmorphism, including mid-face hypoplasia, laryngotracheal cleft, prognathism, cleft palate, bifid epiglottis, micrognathia, and other laryngeal malformations, may lead to difficult intubation or ventilation in these infants [16].

## Conclusions

The care of infants with CDH is fraught with challenges and high perioperative morbidity despite advances in prenatal diagnosis, utilization of protective lung ventilation strategies, and aggressive management of pulmonary hypertension. An infant with PKS exhibits dysmorphic facial features and multisystemic organ involvement that present a host of anesthetic implications. Although the literature has shown a strong association between PKS and CDH, the management of an infant with LV hypoplasia has not been explicitly elucidated previously and provides an added layer of complication to an already challenging clinical case. It is imperative that the anesthesiologist be aware of the complex interplay of CDH and PKS pathophysiology to provide safe anesthetic care to these high-risk infants.

## References

[1] Burgos CM, Frenckner B (2017). Addressing the hidden mortality in CDH: a population-based study. J Pediatr Surg.

[2] Chatterjee D, Ing RJ, Gien J (2020). Update on congenital diaphragmatic hernia. Anesth Analg.

[3] Pierro M, Thébaud B (2014). Understanding and treating pulmonary hypertension in congenital diaphragmatic hernia. Semin Fetal Neonatal Med.

[4] Siebert JR, Haas JE, Beckwith JB (1984). Left ventricular hypoplasia in congenital diaphragmatic hernia. J Pediatr Surg.

[5] Schwartz SM, Vermilion RP, Hirschl RB (1994). Evaluation of left ventricular mass in children with left-sided congenital diaphragmatic hernia. J Pediatr.

[6] Kinsella JP, Steinhorn RH, Mullen MP (2018). The left ventricle in congenital diaphragmatic hernia: implications for the management of pulmonary hypertension. J Pediatr.

[7] Shieh HF, Wilson JM, Sheils CA (2017). Does the ex utero intrapartum treatment to extracorporeal membrane oxygenation procedure change morbidity outcomes for high-risk congenital diaphragmatic hernia survivors?. J Pediatr Surg.

[8] Gien J, Meyers ML, Kinsella JP (2018). Assessment of carina position antenatally and postnatally in infants with congenital diaphragmatic hernia. J Pediatr.

[9] Snoek KG, Capolupo I, van Rosmalen J (2016). Conventional mechanical ventilation versus high-frequency oscillatory ventilation for congenital diaphragmatic hernia. A randomized clinical trial (The VICI-trial). Ann Surg.

[10] Petroze RT, Puligandla PS (2019). Preoperative cardiopulmonary evaluation in specific neonatal surgery. Semin Pediatr Surg.

[11] Leininger K, Chiu K (2022). Anesthetic Considerations In Congenital Diaphragmatic Hernia. https://www.ncbi.nlm.nih.gov/books/NBK572077/.

[12] Logan JW, Cotten CM, Goldberg RN, Clark RH (2007). Mechanical ventilation strategies in the management of congenital diaphragmatic hernia. Semin Pediatr Surg.

[13] Patel N (2012). Use of milrinone to treat cardiac dysfunction in infants with pulmonary hypertension secondary to congenital diaphragmatic hernia: a review of six patients. Neonatology.

[14] Kostanecka A, Close LB, Izumi K, Krantz ID, Pipan M (2012). Developmental and behavioral characteristics of individuals with Pallister-Killian syndrome. Am J Med Genet A.

[15] Schinzel A (1991). Tetrasomy 12p (Pallister-Killian syndrome). J Med Genet.

[16] Cruz JR, Videira RL (2004). Anesthesia in child with Pallister-Killian syndrome: case report [Article in Portuguese]. Rev Bras Anestesiol.

